# Relationship Between Medication Literacy and Medication Adherence in Inpatients With Coronary Heart Disease in Changsha, China

**DOI:** 10.3389/fphar.2019.01537

**Published:** 2020-01-15

**Authors:** Feng Zheng, Siqing Ding, Lin Lai, Xiaoqing Liu, Yinglong Duan, Shuangjiao Shi, Zhuqing Zhong

**Affiliations:** ^1^ Department of Cardiology, The Third Xiangya Hospital of Central South University, Changsha, China; ^2^ Department of Nursing, The Third Xiangya Hospital of Central South University, Changsha, China

**Keywords:** medication safety, medication adherence, medication literacy, inpatients, coronary heart disease, relationship

## Abstract

**Background:** Medication literacy may be associated with medication safety, and medication adherence is critical in treating coronary heart disease. Few studies have explored the association between medication literacy and medication adherence in patients with coronary heart disease. The aim was to investigate the status of medication literacy and medication adherence among Chinese inpatients with coronary heart disease, and explore the association between medication literacy and medication adherence.

**Methods:** The study was a cross-sectional survey. Four hundred seventy inpatients with coronary heart disease were recruited from hospitals in Changsha, Hunan, China. Participants’ demographic and clinical data were retrieved from hospital charts. Medication adherence was assessed using the four-item Morisky Medication Adherence Scale. Medication literacy was assessed using the Chinese Version of the Medication Literacy Scale. For univariate analysis, potential factors influencing medication adherence were tested by T-tests, analysis of variance, and the Kruskal–Wallis H test. Binary logistic regression model was conducted with medication adherence as the outcome variable in order to analyze the association between medication literacy and medication adherence in inpatients with coronary heart disease.

**Results:** Among 512 participants, 470 (91.8%) produced valid responses for the survey. Mean (SD) of medication adherence score was 2.26 (13.6); only 13.6% had optimal medication adherence. Mean (SD) of medication literacy score was 7.52 (4.09); participants with adequate medication literacy was 30.2% (142). Binary logistic regression analysis indicated that medication literacy was an independent predictor associated with medication adherence. Participants with adequate medication literacy were more likely to have optimal medication adherence (OR 1.461 [95% CI: 0.114, 0.643]; P = 0.005), and participants with a high level of education (OR 0.613 [95% CI: 0.284, 0.694]; P< 0.001), a fewer number of medicines (OR 1.514 [95% CI: -0.631, -0.198]; P < 0.001), having medical insurance (OR 0.770 [95% CI: -1.769, 0.059]; P = 0.043), and single inpatients were more likely to be adherent (OR 1.655 [95% CI:-0.858, -0.149]; P = 0.005).

**Conclusions:** The study indicates a significant association between medication literacy and medication adherence in patients with coronary heart disease. These results suggest that medication literacy is an important consideration in the development, implementation, and evaluation of medication adherence interventions.

## Introduction

People with coronary heart disease often require oral medication to achieve and maintain effective symptom control and prevent disease progression (Huang and Chen, 2014; Lam and Fresco, 2015). Not taking medications as prescribed, however, is common. Studies have shown that an estimated 21%–50% of patients with cardiovascular diseases do not adhere to their medication regimens (Wang et al., 2010), and treatment rates among coronary heart disease in China were only 10.6%, 10.1%, 7.6%, and 1.4%, respectively, for anti-platelet drugs, β-blockers, angiotensin-converting enzyme inhibitors (ACEIs)/angiotensin receptor antagonists (ARB), and statins (Chen et al., 2014). Not taking medications as directed has been linked to re-hospitalization and premature mortality (Partin, 2006). Medication adherence plays a critical role in the safety of patients with coronary heart disease (Huang and Chen, 2014; Lam and Fresco, 2015). A cross-sectional study has indicated a positive influence of medication adherence on employees with the following chronic disease—coronary heart disease, diabetes, hypertension, dyslipidemia, and asthma/chronic obstructive pulmonary disease in minimizing losses due to absenteeism and short-term disability caused by medication nonadherence (Carls et al., 2012).

Medication adherence specifically refers to prescribed drug therapy follow-up (Martins et al., 2017). Apart from access to treatment, successful medication adherence requires proper self-administration (Nandyala et al., 2018). The tasks associated with the use of medications are complex (Youmans and Schillinger, 2003; Raynor, 2009). In order to properly use their medications, patients must read the related medical information, including medication labels, instructions, and so on, and take the accurate dose. Individuals also need to decide what actions should be taken to deal with an error dose (e.g., double up or skip it) and potential side effects. These tasks require sufficient medication literacy.

Medication literacy was first mentioned in 2005 in a government document in the United Kingdom addressing quality improvements in providing medication information to individuals with low health literacy (Raynor, 2008). Medication literacy is defined as “the degree to which individuals can obtain, comprehend, communicate, calculate and process patient-specific information about their medications to make informed medication and health decisions in order to safely and effectively use their medications, regardless of the mode by which the content is delivered (e.g. written, oral and visual)” (Pouliot et al., 2017). It can serve as an important predictor of rational medication use (Raynor, 2008; Cordina et al., 2018). Some studies have shown that a large proportion of medication-related adverse events would be preventable with better medication literacy (Zhong et al., 2016; Lee et al., 2017; Zheng et al., 2017). However, the associations between medication literacy and medication adherence have not been clearly explained.

Understanding the role of medication literacy in taking medications may inform efforts to encourage patients take their medication properly and, in turn, could affect clinical outcomes control. There is a lack of correlation studies between medication adherence and medication literacy in patients with chronic disease, and little is known about how medication literacy and medication adherence influence one another. Thus, this study aims to explore the relationship between medication adherence and medication literacy in patients with chronic disease. Our study may offer novel approaches for healthcare providers to target patients and problem areas that require specific interventions to improve medication adherence in patients with chronic disease.

## Materials and Methods

### Design, Setting, and Participants

Upon the approval of the Ethics Review Board of the Third Xiangya Hospital of Central South University (2018-S284), a cross-sectional survey was conducted at three tertiary hospitals (each with >500 inpatient beds) in Changsha, Hunan, China from November 2018 to January 2019. A simple random clustering sampling method was applied. Subjects were eligible if they met the following inclusion criteria: (1) inpatients diagnosed with coronary heart disease by coronary angiography; (2) older than age 18 with competent communication ability; (3) currently taking at least one medication; and (4) mentally stable. Patients were excluded if they had any of the following conditions: (1) engaged in health-care related work currently or before retirement; (2) suffering from a major mental disorder; (3) New York Heart Association class III or IV heart failure; and (4) unwilling to participate in the survey.

### Survey Procedures

Inpatients completed questionnaires anonymously after giving signed informed consent. The survey was carried out by face-to-face interview using paper-and-pencil method. All interviewers were trained master’s degree students majoring in medicine or nursing. Interviews were performed separately for each participant in order to avoid interference. For illiterate subjects, the interviewers read the items word by word exactly, and subjects’ responses were recorded on the questionnaires. The questionnaires were collected immediately after being completed, checked for any missing information, and followed up with the participants.

### Data Collection

Patient’s demographic and clinical data were retrieved from hospital charts, including age, gender, employment status, education, marital status, income, number of health problems, and number of medicines being taken.

### Chinese Version of the Medication Literacy Scale

The Chinese version of the Medication Literacy Scale is a tool to assess medication literacy (Zheng et al., 2016). This scale was originally developed by Sauceda et al. (2012) from the University of Texas at El Paso in the United States. After we obtained permission for its use from Sauceda et al., the English version of the Medication Literacy Scale (MedLitRxSE- English) was translated and adapted for Chinese use, with good reliability (the Kuder-Richardson Formula 20 = 0.81).The validity and reliability of the Chinese version of the MedLitRxSE has been published elsewhere (Zheng et al., 2016). The scale contains four fictitious cases, with 14 items that have a dichotomy scoring system (correct, 1; incorrect, 0). The full score of this scale is 14. According to the principle of calculation formula of the distinction degree, which is used in the educational statistics, the scores were divided into three groups: adequate literacy (> 10), marginal literacy (4–10), and inadequate literacy (< 4). Therefore, the higher the score, the higher the level of medication literacy. The test–retest reliability of the Chinese version for Medication Literacy Scale was 0.885; the split reliability was 0.840; K-R was 0.820; the correlations between the assessment of medication literacy and the corresponding items were 0.427–0.587; the confirmatory factor analysis revealed overall good fit. Root mean square error of approximation (RMSEA), χ2/df, goodness of fit index (GFI), and comparative fit index (CFI) were 0.08, 3.06, 0.91, and 0.94, respectively (Zheng et al., 2016). The Chinese version for the assessment of medication literacy is in good reliability and validity, and it can be used to evaluate the medication literacy.

### Morisky Medication Adherence Scale (MMAS-4)

The four-item Morisky Medication Adherence Scale (MMAS-4) was used to measure the medication adherence (Morisky et al., 1986). The original scale has four items with dichotomous response categories of either yes or no. The questions are negatively coded, with a score of 1 given to a “yes” response and a score of 0 given to a “no” response. Therefore, the lower the score, the better the adherence (0 = high adherence, 1–2 = medium adherence, 3–4 = low adherence) (Morisky et al., 1986). The convergent validity of the four-item Morisky Medication Adherence Scale (MMAS-4) was assessed using Pearson’s correlation, which showed a correlation of 0.6851 (p < 0.01). The Cronbach’s α of the scale is 0.61, supporting its internal consistency reliability (Morisky et al., 1986).

### Statistical Analysis

Analysis was conducted using SPSS (version 17.0, Chicago, U.S.) software. A p-value of less than 0.05 was considered statistically significant. Descriptive statistics were used to describe the inpatient’s baseline characteristics and medication literacy level. For univariate analysis, potential factors influencing medication adherence were tested by T-tests, analysis of variance, and the Kruskal-Wallis H test. Participants with low and medium adherence (1–2 = medium adherence, 3–4 = low adherence) were classified as poor medication adherers, and those with high adherence (0 = high adherence) were classified as good medication adherers. Binary logistic regression analysis was conducted with medication adherence as the outcome variable in order to analyze the association between medication literacy and medication adherence in inpatients with coronary heart disease.

## Results

### General Characteristics of Participants

In the three chosen hospitals, we initially identified 512 eligible inpatients, among whom 28 refused to participate, and 14 were further deleted in data cleaning stage because of missing information on important variables. Therefore, a total of 470 inpatients were analyzed, and the valid response rate is 91.8%. Major characteristics of study subjects are presented in **Table 1**. Three hundred and four patients were male and 166 were female, 96 patients had full-time employment, 442 patients had medical insurance, and 397 patients were married. Means (SDs) of patient age were 60.39 years [standard deviation (SD) **=** 13.09]; the number of medicines that the patient were taking was 5.4 (SD = 1.1).

**Table 1:** Characteristics of the inpatients with coronary heart disease, Changsha, Hunan, China (N=470).

Variable name	No. of participants (N = 470)	Percentage (%)
Age (years)		
18-45	63	13.4
46-55	64	13.6
56-65	156	33.2
>65	187	39.8
Gender		
Male		
Female		
Education (years)		
≤6	150	31.9
7-9	185	39.4
10-12	75	16.0
≥13	60	12.7
Annual household income (in CNY)*		
<11300	219	46.6
11300-30000	161	34.3
>30000	90	19.1
Marital status		
Married	397	84.5
Divorced or widowed	58	12.3
Single	15	3.2
Employment		
Employed	124	26.4
Retired	245	52.1
Unemployed	101	21.5
Insurance		
Yes	442	94.0
No	28	6.0
Number of health problems		
1	251	53.4
2	148	31.5
≥3	71	15.1
Number of medicines currently taken		
≤ 3	112	23.8
4-5	219	46.6
≥6	139	29.6

*¥1=US$0.1456.

### Medication Literacy of Inpatients With Coronary Heart Disease

Details of medication literacy for 470 inpatients with coronary heart disease are displayed in **Table 2**. The mean score of medication literacy was 7.52 (4.09). Patients obtained a score >10 was 30.2% (142) and were considered as “adequate medication literacy”; Those obtained a score 4–10 was 49.8% (234) and were considered as “marginal medication literacy,” whereas 20.0% (94) obtained a score <4 and were considered as “inadequate medication literacy.” For Case Scenario 1, according to the instruction, 41.7% of patients knew the three parts of the body their mother should inject the medicine and 49.4% of patients did not know the right angle. For Case Scenario 2, less than half (40.3%, 48.3%) knew how much dosing of the medicine and the maximum dosage in 1 day their niece should take. For Case Scenario 3, more than half of the patients knew the name and the dosing of the medicine they should buy. For Case Scenario 4, 48.7% of the patients knew the reason they needed to stop the medicine.

**Table 2:** Medication Literacy for Inpatients With Coronary Heart Disease, Changsha, Hunan, China (N = 470).

Item		Correct answers, percentage (%)
Case Scenario 1		
a1	According to the label, how many times one day should your mother inject the medicine?	58.9
a2	Please show me how much medicine you should put into the syringe in the morning, and mark on the Syringe.	53.8
a3	According to the instruction, please tell us or point out where the three parts of the body your mother could inject the medicine?	41.7
a4	According to the instruction, please tell me what is the right angle you should inject the medicine?	50.6
a5	Looking at the prescription, if your mother’s medicine is run out, from whom you should get a new prescription?	55.7
Case Scenario 2		
a6	Looking at the instructions on this box, how much dosing of the medicine should you give to your niece?	40.6
a7	If you know the dosage of medicine that your niece need to take, please mark down on the cup to what line you poured medicine.	50.6
a8	According to the directions, what is the maximum dosage should your niece take?	48.3
Case Scenario 3		
a9	Looking at this prescription, what is the name of the medicine that you need to buy at the pharmacy?	64.9
a10	According to the prescription, how many pills should you take?	60.6
a11	Looking at this bottle, the medicine in the bottle has the similar purpose with the medicine on the prescription, if you need to take 30 pills to cure the infection, how many boxes should you buy to have the correct amount of antibiotic required by the original prescription?	61.7
Case Scenario 4		
a12	Looking at the box, when does the medicine go out of date?	58.7
a13	According to the directions, what is or what are the active ingredients in each pill?	67.7
a14	Please look carefully of the box, for what reason should you stop the medicine?	48.7

### Medication Adherence of Inpatients With Coronary Heart Disease

The result of medication adherence is shown in **Table 3**. Of the 470 participants, mean (SD) of medication adherence score was 2.26 (13.6). Medication adherence was high in 64 participants (13.6%), medium in 208 (44.3%), and low in 198 (42.1%). Around 50% (234) of patients ever forgot to take their medication. More than 50% (244) were careless at times about taking their medicines; 153 (32.6%) participants revealed that they stopped taking their medicines when they felt bad, and 109 (23.2%) reported that they sometimes stopped taking their medicines when they felt better.

**Table 3:** Medication adherence for inpatients with coronary heart disease, Changsha, Hunan, China(N = 470).

Items	No. of participants answered “yes”	Percentage (%)
1.^a^Do you ever forget to take your medicine?	234	49.7
2.^a^Are you careless at times about taking your medicine?	244	51.9
3.^a^When you feel better do you sometimes stop taking your medicine?	153	32.6
4.^a^Sometimes if you feel worse when you take the medicine, do you stop taking it?	109	23.2
Distribution of scores		
High adherence = 0	64	13.6
Medium adherence = 1-2	208	44.3
Low adherence = 3-4	198	42.1

^a^Negatively worded question.

### Associated Factors of Medication Adherence

**Table 4** presents the results of univariate analysis of medication adherence among inpatients with coronary heart disease. Eight factors were significantly associated with medication adherence. Compared to patients with optimal medication adherence, suboptimally adherent patients had lower medication literacy (p-value < 0.001). Optimal medication adherence was found in patients with coronary heart disease who were male (p-value = 0.005), younger (p-value < 0.001), highly educated (p-value < 0.001), single (p-value < 0.001), and those with medical insurance (p-value = 0.001), fewer number of medications (p-value < 0.001), and fewer number of health problems (p-value = 0.001).

**Table 4:** Results of univariate analysis of determinants of medication adherence for inpatients with coronary heart disease Changsha, Hunan, China (N=470).

Variable	Medication adherence		*t/F/H*	*P*
	Mean	SD		
Gender				
Male	2.14	1.28	-2.837*	0.005
Female	2.48	1.20		
Age (years)				
18-45	1.52	1.18	11.193***	<0.001
46-55	1.70	1.17		
56-65	2.32	1.18		
>65	2.64	1.26		
Employment status				
Full-time	1.74	1.14	2.469***	0.064
Retired	2.17	1.34		
Unemployed	2.58	1.28		
Education(years)				
≤6	2.94	1.18	19.215***	<0.001
7-9	2.25	1.09		
10-12	1.64	1.15		
≥13	1.26	1.04		
Marital status				
Single	1.60	0.98	11.491***	<0.001
Married	2.16	1.24		
Divorced or widowed	3.10	1.11		
Medical insurance				
Yes	2.21	1.25	-3.385*	0.001
No	3.04	1.13		
Annual household income (in CNY)				
<11300	2.25	1.08	3.249**	0.197
11300-30000	2.38	1.30		
>30000	2.08	1.54		
Number of health problems				
1	2.05	1.28	7.619***	0.001
2	2.47	1.20		
≥3	2.56	1.18		
Number of medicines currently taken				
≤ 3	1.98	1.25	16.635***	<0.001
4-5	2.52	1.19		
≥6	2.82	0.97		
Medication literacy				
Inadequate (<4)	2.66	1.46	109.066**	<0.001
Marginal (4–10)	2.64	1.06		
Adequate (>10)	1.37	0.94		

¥1 =US$0.1445; t (*), two-sample t-test; H (**), Kruskal–Wallis test; F (***), analysis of variance.

**Table 5** shows the results of binary logistic regression analysis of medication adherence among patients with coronary heart disease. Five factors that showed an independent association with medication adherence are as follows: medication literacy (OR 1.461 [95% CI: 0.114, 0.643]; P = 0.005), education level (OR 0.613 [95% CI: 0.284,0.694]; P< 0.001), number of medicines (OR 1.514 [95% CI: -0.631,-0.198]; P < 0.001), medical insurance (OR 0.770 [95% CI: -1.769,0.059]; P = 0.043), and marital status (OR 1.655 [95% CI:-0.858,-0.149]; P = 0.005). Medication adherence increased with an adequate medication literacy level, a high level of education, a fewer number of medicines, and having medical insurance. For marital status, divorced or widowed patients were less likely to be adherent.

**Table 5:** Results of binary logistic regression analysis of determinants of medication adherence for inpatients with coronary heart disease Changsha, Hunan, China (N = 470).

Effect	b	Sb	P	Odds ratio	95% CI
Medication literacy	–0.742	0.164	0.005	1.461	(0.114, 0.643)
Education (years)	–0.489	0.105	<0.001	0.613	(0.284, 0.694)
Number of medicines currently taken	0.415	0.110	<0.001	1.514	(-0.631, -0.198)
Marital status	0.504	0.181	0.005	1.655	(-0.858, -0.149)
Medical insurance	–0.261	0.129	0.043	0.770	(-1.769, 0.059)

b, partial regression coefficient; Sb, standard error; 95% CI, 95% confidence intervals.

## Discussion

In this population-based cross-sectional study, we described the status of medication adherence and medication literacy and explored possible influencing factors of medication adherence in a group of Chinese inpatients with coronary heart disease. Based on analytical results, we found that, of the 470 participants, mean (standard deviation) of medication adherence score was 2.26 (13.6). Patients with high adherence was 13.6%, 44.3% had medium adherence, and 42.1% had low adherence. The mean (standard deviation) of medication literacy score was 7.52 (4.09). Medication literacy was adequate in 142 participants (30.2%), marginally adequate in 234 (49.8%), and inadequate in 94 (20.0%). The reported medication adherence in our study was suboptimal and low compared to the global range (Mosleh and Almalik, 2016; Kim et al., 2016; Mondesir et al., 2018). It was also lower than 68.9%–84.7% from previous studies carried out in China or other countries (Mondesir et al., 2018; Lu et al., 2019).

This difference may be explained by the difference in health care settings, patients’ knowledge, health literacy, complexity of patients’ regimens, socioeconomic status, sampling methods, and/or metrics used for adherence assessment among researches. In this study, in order to recruit a more representative study population, various districts were included, whereas the previous study in China was done in a single center. This may indicate that the actual situation of medication adherence in China was more worrying than the previous researches had estimated. In the same time, we found that patients with inadequate medication literacy was higher than those reported from some previous studies (Zhong et al., 2016; Zheng et al., 2017), though different populations were studied and various scales were used for medication literacy measure. This indicated that medication literacy of the patients has further room for improvement.

Our study showed that patients’ medication literacy had been recognized as a factor affecting medication adherence according to logistic regression analysis. The possible reason is that patients with higher medication literacy may have better understanding towards regimes and be skillful to take medicine, hence reinforcing their medication adherence (Raynor, 2009). Medication literacy is the manifestation of health literacy in the context of medication use, and inappropriate medication use was identified to be significantly associated with low medication literacy level (Kripalani et al., 2008; Zheng et al., 2017; Lee et al., 2017). Although medication literacy is assumed to be related to health literacy, medication literacy needs more skills to practice (Sauceda et al., 2012; Pouliot et al., 2017; Yeh et al., 2017). Some studies have shown that health literacy has an insignificant and weakly positive association with self-reported medication adherence (Zhang et al., 2014; Sawkin et al., 2015; Park et al., 2018), but the findings in our study suggested that medication literacy has a significant association with adherence to medication. It may be that the instruments that evaluate health literacy are said to be predictors of health-related behaviors (Parker et al., 1995). These tools focus on reading comprehension and numeracy, but not on the action of interpreting health information (Parker et al., 1995). A medication literacy assessment tool was used to assess medication literacy level (Sauceda et al., 2012). It involves specific skills that are not adequately assessed using general health literacy assessments. So our result is significant, suggesting that it may not be sufficient to only consider general factors; we should also take medication literacy into account to ensure appropriate medication adherence among patients with coronary heart disease with limited medication literacy, and to decrease the risk of worse health outcomes. Many ways to achieve it could be through increasing patients’ awareness about proper medication use, improving communication between patients and health care providers, meeting with a cardiovascular educator on a regular basis, using educational materials, and establishing a community campaign.

Besides medication literacy, education (years), number of medicines, marital status, and medical insurance were reported to be the significant determinants by logistic regression. The results are consistent with the findings of previous studies (Brown and Bussell, 2011; Curkendall et al., 2013; Rolnick et al., 2013). Therefore, an integrated multifaceted step is needed to improve medication adherence in patients with chronic disease (Williams et al., 2008).

The study’s strengths are as follows. The findings showed that medication literacy is related to medication adherence, whereas most of the previous studies concentrated on the relationship between health literacy and medication adherence. The results of this study will raise awareness of the impact of medication literacy on medication safety. Additionally, a validated assessment was used to assess medication literacy. There are some limitations in this study. First, the information on medication adherence largely depended on patients’ recall, which could not be completely accurate. Second, this study was carried out in one city of China; our results may not be representative. More larger sample and more areas of studies should be conducted. In addition, our study is a cross-sectional design and thus cannot allow causal relationships to be concluded among the variables.

## Conclusion and Implications

Our findings indicate a significant association between medication literacy and medication adherence in patients with coronary heart disease. The level of medication literacy and medication adherence among patients with coronary heart disease is suboptimal and needs to be improved. Our study provides preliminary information for developing effective healthcare intervention programs.

## Data Availability Statement

All datasets generated for this study are included in the article.

## Ethics Statement

The studies involving human participants were reviewed and approved by the ethics committee of the Third Xiangya Hospital of Central South University. The patients/participants provided their written informed consent to participate in this study.

## Author Contributions

ZZ conceived the study. SD, YD, LL, SS, and XL collected, verified, and analyzed the data. FZ drafted the manuscript. All authors provided critical revision of the manuscript for important intellectual content.

## Funding

This work was supported by the Natural Science Foundation Project of Hunan Province, China, under Grant No. 2019JJ50905 and the Natural Science Foundation Project of China, under Grant No. 71603290.

## Conflict of Interest

The authors declare that the research was conducted in the absence of any commercial or financial relationships that could be construed as a potential conflict of interest.

## References

[B1] Brown M. T. Bussell J. K. (2011). Medication adherence: who cares? Mayo. Clin. Proc. 86 (4), 304–314. 10.4065/mcp.2010.0575 21389250 PMC3068890

[B2] Carls G. S. Roebuck M. C. Brennan T. A. Slezak J. A. Matlin O. S. Gibson T. B. (2012). Impact of medication adherence on absenteeism and short-term disability for five chronic diseases. J. Occup. Environ. Med. 54 (7), 792–805. 10.1097/JOM.0b013e31825463e9 22796923

[B3] Chen Y. Li L. Zhang Q. Clarke R. Chen J. Guo Y. (2014). Use of drug treatment for secondary prevention of cardiovascular disease in urban and rural communities of China: China Kadoorie Biobank Study of 0.5 million people. Int. J. Cardiol. 172 (1), 88–95. 10.1016/j.ijcard.2013.12.065 24461961 PMC3991854

[B4] Cordina M. Hämeen-Anttila K. Lauri J. Tabon S. Enlund H. (2018). Health and medication literacy and the desire to participate in pharmacotherapy decision making - comparison of two countries. Res. Social. Adm. Pharm. 14 (9), 817–823. 10.1016/j.sapharm.2018.06.009 29934278

[B5] Curkendall S. M. Thomas N. Bell K. F. Juneau P. L. Weiss A. J. (2013). Predictors of medication adherence in patients with Type 2 diabetes mellitus. Curr. Med. Res. Opin. 29 (10), 1275–1286. 10.1185/03007995.2013.821056 23815104

[B6] Huang J. Y. Chen H. M. (2014). Concept analysis of medication adherence in patients with chronic disease. J. Nursing. 61 (3), 112–118. 10.6224/JN.61.3.112 24899565

[B7] Kim S. Shin D. W. Yun J. M. Hwang Y. Park S. K. Ko Y. J. (2016). Medication adherence and the risk of cardiovascular mortality and hospitalization among patients with newly prescribed antihypertensive medications. Hypertension 67 (3), 506–512. 10.1161/HYPERTENSIONAHA.115.06731 26865198

[B8] Kripalani S. Henderson L. E. Jacobson T. A. Vaccarino V. (2008). Medication use among inner-city patients after hospital discharge: patient-reported barriers and solutions. Mayo. Clin. Proc. 83 (5), 529–535. 10.4065/83.5.529 18452681

[B9] Lam W. Y. Fresco P. (2015). Medication adherence measures: an overview. Biomed. Res. Int. 217047, 1–12. 10.1155/2015/217047 PMC4619779 26539470

[B10] Lee C. H. Chang F. C. Hsu S. D. Chi H. Y. Huang L. J. Yeh M. K. (2017). Inappropriate self-medication among adolescents and its association with lower medication literacy and substance use. Plos One 12 (12), e0189199. 10.1371/journal.pone.0189199 29240799 PMC5730183

[B11] Lu M. Ma J. Lin Y. Zhang X. Shen Y. Xia H. (2019). Relationship between patient’s health literacy and adherence to coronary heart disease secondary prevention measures. J. Clin. Nurs. 28 (15–16), 1–11. 10.1111/jocn.14865 30938879

[B12] Martins N. F. F. Abreu D. P. G. Silva B. T. D. Semedo D. S. D. R. C. Pelzer M. T. Ienczak F. S. (2017). Functional health literacy and adherence to the medication in older adults: integrative review. Rev. Bras. Enferm. 70 (4), 868–874. 10.1590/0034-7167-2016-0625 28793120

[B13] Mondesir F. L. Carson A. P. Durant R. W. Lewis M. W. Safford M. M. Levitan E. B. (2018). Association of functional and structural social support with medication adherence among individuals treated for coronary heart disease risk factors: findings from the reasons for geographic and racial differences in stroke (REGARDS) study. PLoS One 13 (6), e0198578. 10.1371/journal.pone.0198578 29949589 PMC6021050

[B14] Morisky D. E. Green L. W. Levine D. M. (1986). Concurrent and predictive validity of a self-reported measure of medication adherence. Med. Care 24 (1), 67–74. 10.1097/00005650-198601000-00007 3945130

[B15] Mosleh S. M. Almalik M. M. (2016). Illness perception and adherence to healthy behaviour in Jordanian coronary heart disease patients. Eur. J. Cardiovasc. Nurs. 15 (4), 223–230. 10.1177/1474515114563885 25505161

[B16] Nandyala A. S. Nelson L. A. Lagotte A. E. Osborn C. Y. (2018). An analysis of whether health literacy and numeracy are associated with diabetes medication adherence. Health Lit. Res. Pract. 2 (1), e15–e20. 10.3928/24748307-20171212-01 30112462 PMC6088806

[B17] Park N. H. Song M. S. Shin S. Y. Jeong J. H. Lee H. Y. (2018). The effects of medication adherence and health literacy on health-related quality of life in older people with hypertension. Int. J. Older People Nurs. 13 (3), e12196. 10.1111/opn.12196 29665241

[B18] Parker R. M. Baker D. W. Williams M. V. Nurss J. R. (1995). The test of functional health literacy in adults: a new instrument for measuring patients’ literacy skills. J. Gen. Intern. Med. 10 (10), 537–541. 10.1007/bf02640361 8576769

[B19] Partin B. (2006). Preventing medication errors: an IOM report. Nurse. Pract. 31 (12), 8. 10.1097/00006205-200612000-00002 17149128

[B20] Pouliot A. Vaillancourt R. Stacey D. Suter P. (2017). Defining and identifying concepts of medication literacy: an international perspective. Res. Soc. Adm. Pharm. 14 (9), 797–804. 10.1016/j.sapharm.2017.11.005 29191647

[B21] Raynor D. K. (2008). Medication literacy is a 2-way street. Mayo. Clinic. Proc. 83 (5), 520–522. 10.4065/83.5.520 18452678

[B22] Raynor D. K. (2009). Addressing medication literacy: a pharmacy practice priority. Int. J. Pharm. Pract. 17 (5), 257–259. 10.1211/ijpp/17.05.0001 20214266

[B23] Rolnick S. J. Pawloski P. A. Hedblom B. D. Asche S. E. Bruzek R. J. (2013). Patient characteristics associated with medication adherence. Clin. Med. Res. 11 (2), 64–65. 10.3121/cmr.2013.1113 PMC3692389 23580788

[B24] Sauceda J. A. Loya A. M. Sias J. J. Taylor T. Wiebe J. S. Rivera J. O. (2012). Medication literacy in Spanish and English: psychometric evaluation of a new assessment tool. J. Am. Pharm. Assoc. 52 (6), e231–e240. 10.1331/JAPhA.2012.11264 23229985

[B25] Sawkin M. T. Deppe S. J. Thelen J. Stoner S. C. Dietz C. A. Rasu R. S. (2015). Health literacy and medication adherence among patients treated in a free health clinic: a pilot study. Health Serv. Res. Manage. Epiemiol. 2, 1–7. 10.1177/2333392815589094 PMC5266426 28462257

[B26] Wang F. X. Xu W. F. Hua T. T. Zhou D. J. (2010). Sampling investigation of the situation of rational drug use in China. China Pharmacy. 21 (17), 1541–1544. 1001-0408201017-1541-04

[B27] Williams A. Manias E. Walker R. (2008). Interventions to improve medication adherence in people with multiple chronic conditions: a systematic review. J. Adv. Nurs. 63 (2), 132–143. 10.1111/j.1365-2648.2008.04656.x 18537843

[B28] Yeh Y. C. Lin H. W. Chang E. H. Huang Y. M. Chen Y. C. Wang C. Y. (2017). Development and validation of a Chinese medication literacy measure. Health Expect. 20 (6), 1296–1301. 10.1111/hex.12569 28474423 PMC5689244

[B29] Youmans S. L. Schillinger D. (2003). Functional health literacy and medication use: the pharmacist’s role. Ann. Pharmacother. 37 (11), 1726–1729. 10.1345/aph.1D070 14565798

[B30] Zhang N. J. Terry A. Mchorney C. A. (2014). Impact of health literacy on medication adherence: a systematic review and meta-analysis. Ann. Pharmacother. 48 (6), 741–751. 10.1177/1060028014526562 24619949

[B32] Zheng F. Zhong Z. Q. Ding S. Q. Luo A. J. Liu Z. N. (2016). Modification and evaluation of assessment of medication literacy. J. Cent. South. Univ. 41 (11), 1226–1231. 10.11817/j.issn.1672-7347.2016.11.019 27932772

[B31] Zheng F. Ding S. Q. Luo A. J. Zhong Z. Q. Duan Y. L. Shen Z. Y. (2017). Medication literacy status of outpatients in ambulatory care settings in Changsha, China. J. Int. Med. Res. 45 (1), 303–309. 10.1177/0300060516676726 PMC5536586 28222647

[B33] Zhong Z. Q. Zheng F. Guo Y. N. Luo A. J. (2016). Medication literacy in a cohort of Chinese patients discharged with acute coronary syndrome. Int. J. Environ. Res. Public Health 13 (7), 720. 10.3390/ijerph13070720 PMC4962261 27428990

